# Desired Features of a Digital Technology Tool for Self-Management of Well-Being in a Nonclinical Sample of Young People: Qualitative Study

**DOI:** 10.2196/10067

**Published:** 2018-12-18

**Authors:** Camilla Babbage, Georgina Margaret Jackson, Elena Nixon

**Affiliations:** 1 Division of Psychiatry and Applied Psychology School of Medicine University of Nottingham Nottingham United Kingdom

**Keywords:** adolescence, young people, well-being, self-management, digital technology, E-health, coping strategies, mental health, help-seeking, qualitative

## Abstract

**Background:**

Adaptive coping behaviors can improve well-being for young people experiencing life stressors, while maladaptive coping can increase vulnerability to mental health problems in youth and into adulthood. Young people could potentially benefit from the use of digital technology tools to enhance their coping skills and overcome barriers in help-seeking behaviors. However, little is known about the desired digital technology use for self-management of well-being among young people in the general population.

**Objective:**

This is a small, qualitative study aimed at exploring what young people desire from digital technology tools for the self-management of their well-being.

**Methods:**

Young people aged 12-18 years were recruited from the general community to take part in semistructured interviews. Recorded data from the interviews were transcribed and analyzed using inductive thematic analysis.

**Results:**

In total, 14 participants were recruited and completed the study, with a mean age of 14.6 years (female n=3). None of the participants reported using any digital tools specifically designed to manage well-being. However, as indicated through the emerged themes, young people used digital technology to reduce their stress levels and manage their mood, mainly through games, music, and videos. Overall, identified themes showed that young people were keen on using such tools and desired certain facets and features of an ideal tool for self-management of well-being. Themes related to these facets indicated what young people felt a tool should do to improve well-being, including being immersed in a stress-free environment, being uplifting, and that such a tool would direct them to resources based on their needs. The feature-based themes suggested that young people wanted the tool to be flexible and enable engagement with others while also being sensitive to privacy.

**Conclusions:**

The young people interviewed in this study did not report engaging with digital technology specialized to improving well-being but instead used media already accessed in their daily lives in order to self-manage their psychological states. As a result, the variety of coping strategies reported and digital tools used was limited to the resources that were already being used for recreational and social purposes. These findings contribute to the scarce research into young people’s preferred use of digital technology tools for the self-management of their well-being. However, this was a small-scale study and the current participant sample is not representative of the general youth population. Therefore, the results are only tentative and warrant further investigation.

## Introduction

It is estimated that 10-20% of young people experience mental health problems worldwide [1], with 75% of youth being diagnosed with a mental health disorder before the age of 24 [2]. Despite the widely documented reduced well-being levels in youth, there is a lack of research on mental health issues experienced in the general youth population, with most of the literature being focused on youth mental health in clinical groups diagnosed with mood, anxiety, or associated mental health problems [3-9].

There is considerable evidence to suggest that young people’s reduced well-being levels are largely attributed to an inability to cope effectively with stressors stemming from social, physical, and emotionally challenging situations [10,11]. Psychosocial stress, in particular, has been deemed a key factor contributing to high levels of distress in youth, especially during the transitional period from pre-adolescent to adolescent phases when there is increasing accumulation of stressful life experiences, for example, peer, school, and family relationships and events [12,13].

Given that reduced well-being levels in nonclinical youth have been associated with maladaptive coping behaviors [3-9], the use of adaptive coping strategies can play a catalytic role in helping young people manage their stress levels and in reducing the risk of their developing mental health problems in later years [14,15]. Research shows that problem-focused coping strategies (ie, involving directing one’s efforts toward the stressor) can be helpful as they have been associated with positive health outcomes [16]. However, such strategies do not seem to align with the strategies that young people typically use. They frequently adopt either emotion-focused coping techniques in their attempt to regulate their emotions or escape-avoidance strategies by directing their attention away from the problem [11,17,18].

Furthermore, young people tend to show low help-seeking behaviors, often choosing informal offers of support over professional sources of help provision [19-22]. At the same time, mental health services do not have the capacity or resources to sufficiently meet the needs of the young people accessing services [23,24]. In recent years, increased use of digital technology tools has facilitated the provision of health interventions and health-related information through various communication channels and platforms. Self-help digital technology tools have been found to be easily accessible and user-friendly [25] and could therefore help overcome the documented barriers to accessing mental health services [26-28]. However, research into their acceptability by young people is limited.

Findings from a scarce number of studies in nonclinical youth populations have revealed concerns about the use of digital technology tools for the self-management of their well-being, such as the lack of face-to-face support [28-31]. In addition, young people have expressed their desire for these tools to be engaging, interactive, and personable [32], and to provide a variety of online sources of information about self-help on mental health issues rather than directing them to professionals [33]. These findings seem to indicate that the nonclinical youth would find digital technology tools helpful for the management of their well-being if certain desired or disliked tool elements were incorporated or excluded, respectively. To further this understanding, this current qualitative study aimed to explore what features and facets young people would desire from digital technology tools for the purposes of managing their well-being.

## Methods

### Participants and Recruitment

We recruited 14 young people aged 12-18 years from the community via flyers posted on social media, forums, and through gatekeepers to groups and organizations including youth groups (eg, church, community, government, sports, drama, and charities), local schools, and study participation registers. All participants were recruited from the Nottinghamshire region of East Midlands, United Kingdom, and were in Years 10-13 of secondary school education. The criteria for inclusion required that participants be aged 12-18 years and have previous experience in using digital technology. No exclusion criteria were applied. History of experience or clinical diagnosis of mental health problems was not an exclusion criterion, but participants were asked by the researcher to report on current or previous experience of mental health issues.

Participants did not receive any monetary allowance for participating in the research. This research was approved by, and adhered to the guidelines of, the University of Nottingham Division of Psychiatry and Applied Psychology Ethics Board (United Kingdom).

### Study Procedure

Participants contacted the researcher via gatekeepers or directly through the email address provided on the flyers to ask about the study or express their interest in participating in the study. Informed consent forms, and assent forms for participants under 16, were completed electronically. The date and preferred mode of interview (ie, video or voice call) were arranged by email prior to the day of the interview. On the day of the interview, an overview of the study procedure was first provided to participants, reminding them also that they could withdraw from the study at any point and that they should feel free not to answer any questions they felt uncomfortable with. On obtaining verbal consent, in addition to the consent or assent obtained electronically, the researcher proceeded with the interview.

The Mobile Phone Use Survey [34] and previous research into the functions of digital apps [35] helped inform the interview guide and prompts used in the semistructured interviews. Open-ended questions were used to produce in-depth information, followed by a closed or probing format of questioning, where applicable, to elicit further detail (see Multimedia Appendix 1). Interviews lasted approximately 20 minutes. The initial part of the interview was designed to explore what types of digital technology are used by young people as well as young people’s views and feelings about how their well-being may affect or be affected by their use of this technology. The remaining interview questions centered on the desired features and facets of an ideal tool, that is, exploring youth’s preferences for digital tools aimed at assisting them with nonclinical psychological well-being issues.

### Data Analysis

Interview recordings were transcribed verbatim and analyzed using inductive thematic analysis. Express Scribe Transcription software (version 6.0) was used for the organization and development of codes and themes by the researcher (CB). In line with Braun and Clarke’s 6-step recursive process of thematic analysis [36], transcribed interviews were checked against audio recordings for accuracy, then read and re-read by the researcher to ensure familiarization with the data. Following the familiarization stage, initial codes were generated where participants’ responses were relevant to the research question. Codes were subsequently organized into theme categories by CB, which were also reviewed and verified by the co-authors (EN, GMJ) before defining the final themes and subthemes. Both EN and GMJ are experienced in assessing qualitative research. A codebook example can be found in Multimedia Appendix 2, illustrating how themes were generated according to previously proposed codebook guidelines [30,31].

The researcher, CB, is a postgraduate applied psychology student trained in conducting thematic analysis and is conscious that the knowledge gained from thematic analysis and its interpretation is influenced by factors such as the researcher’s previous thoughts on the research subject, cognition, use of language, culture, perceptions, and emotions [36]. To minimize the influence of bias and increase credibility of the research, a self-reflexive approach to the research was used throughout, which included keeping a reflexive journal in line with thematic analysis recommendations [36,37]. These notes were shared with EN and GMJ at the end of the thematic analysis so that they could be reflected on prior to the interpretation of the results.

## Results

### Participant Recruitment

A total of 14 young people aged 12-18 years were recruited and completed the study (mean age 14.6 years, SD 1.6; 3 females). None of the participants reported having a current or previous clinical diagnosis of mental health problems.

Due to the small-scale nature of this study, a sample of 15 young people was intended to be drawn but recruitment stopped at 14 participants because data saturation was reached for the identified key themes. No more depth could have been achieved due to the limited scope of the digital technology used and of the features and facets reported by the sample. In accordance with thematic analysis guidelines and recent reviews, the final sample was sufficiently sized for the purposes of conducting thematic analysis [38,39].

### Overview of Findings

In response to our questions on what young people desire from an ideal digital technology tool designed for managing one’s well-being, none of the participants reported using digital tools specifically designed for the self-management of well-being, such as self-help mobile or Internet apps for managing stress or improving one’s mood. Instead, they tended to refer to the media they would use or expect to use to help them manage their psychological well-being, such as games, music, and platforms for contacting friends. These media were already being used by young people on a daily basis for recreational purposes, but young people reported that they would like to see such media featured in an ideal self-help well-being tool, as well as on other facets and features they would like an ideal tool to incorporate.

The emerged themes concerned the specific facets and features that young people expected to find or desired in a self-help digital technology tool designed to help them improve their well-being. All participants unanimously stated that the applications of such a tool should all be offered on a mobile phone platform due to its accessible and convenient nature. The reported facet-based themes referred to what the ideal tool “should do” in order to improve the young person’s well-being, for example, “The ideal tool should allow oneself to be immersed in a stress-free environment” (Theme 1), “The ideal tool must have an uplifting effect to be helpful” (Theme 2), and “The ideal tool should assess and direct one to resources that match one’s needs” (Theme 3). With regard to the desired features of the tool, young people expressed that, “The ideal tool should be sensitive to privacy” (Theme 4), “Flexibility in choice and resources is a desired feature of the ideal tool” (Theme 5), and “The ideal tool should enable engagement with others” (Theme 6).

### Facet-Based Themes

#### Theme 1 (T1): The Ideal Tool Should Allow Oneself to Be Immersed in a Stress-Free Environment

Young people reported that using music and playing games or talking to others while gaming, on their mobile phones, tablets, and game stations, was a means for them to de-stress.

##### T1:1 A Means of Distraction From Stressful Thoughts

Participants wanted the tool to offer a relief from the stressors they were experiencing by acting as a distractor, for example, using online games as a means of contact with friends and distraction from stressors.

Because distracting would take your mind off the stress and stuff like that as well and so you eventually forget.

P7, 14 years, male

Yeah when I’m stressed out and everything like, like some days I’ve been doing a project and it has been annoying me because it’s quite fiddly and when it’s, most of the time I go on that and I play online games, and you’re playing other people and it makes me feel connected.

P6, 13 years, male

##### T1:2 A Means of Relaxation or Escape

Participants felt that a purpose of the tool should be to enable one to relax, providing relief from the stressor or to remove oneself from the problematic situation as a means of escape from the stressor. Use of music and games were reported as means to relax and reduce stress-related feelings.

I find music to be very umm, again to be very soothing and easing of me. I am a huge fan of the Beatles. Whenever I feel anxious and stressed or angry, or any kind of negative emotion, listening to them really helps me a great deal, it kind of grounds me and stuff.

P3, 18 years, female

Umm but, I’d say games are the biggest things that help me destress, I’d say that’s the main thing. Yeah I sort of forget about where I am in the real world, I can engage myself, sort of help you relax and forget about anything that I’m thinking about in real life.

P3, 14 years, male

#### Theme 2 (T2): The Ideal Tool Must Have an Uplifting Effect to Be Helpful

Young people stated that the tool ought to produce an uplifting effect in order to boost one’s mood in the short-term.

##### T2:1 Use of Videos to Motivate or to Make One Feel Better

Videos were considered to have the ability to improve one’s negative mood, for example when featuring inspirational stories or funny scenes.

Yeah…I think it’s hard raising someone’s mood without being there yourself so I think like more videos or, this, it could just be one video or maybe two, it could just be like, TED talks. I know TED talks do loads of videos about how to cope with that, so it could be more about like self-help and distraction in that sense.

P5, 18 years, female

##### T2:2 Use of Music to Help Regulate Mood

Participants referred to the use of music as one of the preferred means of mood regulation, in order to improve mood but also to help them reflect on their current emotions: “Yeah, like the type of music can reflect on the mood you’re feeling” (P11, 14 years, male).

#### Theme 3 (T3): The Ideal Tool Should Assess and Direct One to Resources That Match One’s Needs

It was anticipated by participants that the ideal tool would “know” how one feels and what would make them feel better and direct them to the appropriate resources. Some of these resources should be available within the tool’s features while others could be resources the tool signposts to.

##### T3:1 Provides Resources to Overcome Negative Emotions

The ideal tool was expected to contain directly supportive functions for dealing with negative emotions. The idea was that after the user informs the tool of their mood, the tool provides the user with information on what to do as well as information about the user’s previously logged preferences.

Like you just tell the app…what you enjoy doing and it kind of picks certain things so you can kind of go, oh get back to what, like if you were stressed out, get back to calm state, just using the app, or tool or whatever and it will kind of use your information that you’ve told it to give you something, like a quiz or something just to feel better.

P2, 15 years, male

##### T3:2 Provides Information and Direction for Further Support

Young people expressed a desire for the tool to enable them to improve their knowledge and direct them to external professional sources for dealing with severe issues around their well-being.

Yeah, so I think if someone’s very often clicking like low mood and a certain aspect maybe if the app could just like come a bit more focused and talk about, not brainwashing, but develop itself so that the videos it shows could be of someone being like, about them getting help and external forces as well, just like, giving these ideas and showing that there are other ways to do it as well and talking and stuff.

P5, 18 years, female

### Feature-Based Themes

#### Theme 4 (T4): The Ideal Tool Should Be Sensitive to Privacy

One of the major concerns young people had about tool use was privacy and exposure of personal data. They had various concerns about social media featuring on the tool and how this would affect their privacy. They also expressed opinions on the degree of parental involvement in the use of the tool.

##### T4:1 Provides Safeguards as Needed to Limit Disclosure of Personal Data

Young people would prefer not to have to enter personal details on the tool or have site-monitoring features on a tool. Particular concerns were raised around exposure of personal information through social media on current digital technology tools.

Yeah, because like on Snapchat now you can see where people are and I just think that’s a bit over the top. Like I’ve put myself on ghost mode now but I didn’t realize I had that until the app updated itself, which was a couple of weeks later, and when I found it made me quite uncomfortable because I feel like everyone on Snapchat knew what I was doing.

P4, 16 years, female

##### T4:2 Parental Involvement Is Acceptable When Necessary

Parental access to the tool was generally not a desired feature, but parental involvement was deemed acceptable when necessary, as in the case of serious risk of self-harm. Young people suggested that the tool should prompt the user to talk to their parents or enable parents to be informed if needed.

#### Theme 5 (T5): Flexibility in Choice and Resources Is a Desired Feature in the Ideal Tool

A crucial element desired of the tool’s features was flexibility and capacity for personalization so that it would be tailored to an individual’s wide range of needs. The main areas identified as necessitating flexibility included the identification and selection of current emotional state, and selection capacity in games, videos, and music.

##### T5:1 Choice for Reflecting Different Emotional States

Participants felt the tool should be able to support different emotional states so that the young person has a wide range of options to select from in order to identify the mood that best reflects their current emotional state: “Yeah, umm I think the main thing that would interest me with a tool like that is if it gave you choice. It depends on how I’m feeling and what exactly I feel I’m in the mood for to do” (P13, 14 years, male).

##### T5:2 Games and Puzzles to Suit Individual Preference

Games were identified as a means of help for managing negative moods and for stress reduction, particularly through distraction. It was deemed necessary for the tool to offer options for different games that suit different people’s tastes in order to enable distraction and hence make one feel better.

It would help if I was stressed out to take my mind off it and you could like choose what you want or what your favorite kind of thing is, if you like puzzles, or like quizzes or games and bits, you can just choose whatever you want, that would help a lot.

P2, 15 years, male

##### T5:3 Videos Provide a Variety of Resources

Videos were highly desired by participants and were expected to be adaptable and engaging resources expected to serve a variety of functions, including mood boosting and information provision.

##### T5:4 Music for Individual Preference

Music was often mentioned to be an ideal means of mood self-management, but the tool would have to be able to select the desired music from a range of options that would fit one’s personal taste.

#### Theme 6 (T6): The Ideal Tool Should Enable Engagement With Others

Communicating with others in order to obtain their support was a highly desired aspect of the tool, but there was a preference for this function to be facilitated through means other than social media.

##### T6:1 Communication With Friends for Connection

Young people expressed the desire for the tool to enable them to contact friends for improving mood and for maintaining friendships, although it was noted that this should take place outside the context of social media.

I mean having the option to open it out to normal people might be quite interesting because it means that if your friends aren’t able to play then you have the option to open it up to more people but there are risks in that on meeting people that you don’t know well. Normally if I have to do something like that, I would only talk to friends.

P13, 14 years, male

##### T6:2 Anonymous Communication for Support

Participants felt that blogs or forums enabling communication with people unknown to the user could offer a type of support that would be different from that offered by friends or other familiar people: “Maybe like blog like a forum, or something, where other people can anonymously put things and ask for advice of other people” (P7, 14 years, male).

## Discussion

### Principal Findings

This study looked at what young people would like to see featured in an ideal digital technology tool designed to help them cope with nonclinical well-being issues. Despite the small sample size and the relatively limited breadth of theme-related content addressing the research question, our findings showed that young people did not use technology specifically targeted at well-being management. Instead, in their attempt to improve their psychological well-being, they tended to use the same digital technology they would use on a daily basis for recreational or social purposes. Although this meant that the reported range of technology use was unexpectedly limited, hence narrowing the scope of the research question, young people’s accounts still provided an interesting insight into the coping strategies that they tended to seek, such as relaxation and distraction. They also provided some evidence in support of young people’s preferences for an ideal self-help well-being tool that would have to be flexible, interactive, and sensitive to privacy.

Consistent with the reported current technology use patterns of the young people included in this study sample, such self-help tools would be preferred to be implemented as apps on mobile phone platforms, which were reported as the most accessible means. Emerged themes denoted a fundamental expectation that the tool will have to fulfil its intended purpose, that is, to improve well-being levels mainly by boosting mood and reducing anxiety levels. Specifically, the expectation that the ideal tool would improve young people’s mood and motivation levels, particularly through use of music and videos, could be considered as an adaptive approach of mood regulation [14,40]. Moreover, young people wanted the tool to enable them to adopt short-term distraction and escape coping techniques for stress relief purposes, mirroring the techniques adopted in their current use of digital technology. This finding suggests a tendency by young people to direct their attention away from the stressor [11,14,18], an approach that can be of adaptive value in the short-term leading to positive mental health outcomes in youth [16,41]. However, continual use of adaptive distraction has been associated with low levels of well-being and poor mental health outcomes more generally [3-5,11,42,43], perhaps because long-term distraction can hinder appropriate action responses [18] and may reinforce the persistence of avoidance-escape approaches [16]. Such a tendency for short-term distraction is in line with previous literature [3-5] and may be indicative of young people’s perceived lack of ability to exert control over a stressful situation [44,45].

In line with the assumption that self-management tools provide a means of overcoming barriers to support seeking in young populations [46-48], another desired facet of an ideal well-being tool was the inclusion of pointers to resources for social support as well as psychoeducation about mental health and illness. Support seeking in this context portrays a problem-focused approach given young people’s desire to be directed to resources that match their needs, reflecting their willingness to turn their attention towards the problem, provided that they know where to turn to for help. Notably, the need for psychoeducation may reflect the documented low mental health literacy in adolescent populations [49] and could have important implications for young people’s proactive use of well-being tools. Easy access to information about mental health problems as well as sources for help could equip young people with the knowledge and skills to help them recognize mental health issues and develop effective strategies to optimize their well-being [50]. Flexibility in function and choice was also expected to be featured in an ideal self-help well-being tool, consistent with previous literature [3-5]. An unforeseen finding in our study was that although young people wanted to engage with others, they were not keen on contacting others through social media, mainly because of concerns around privacy. This apprehension has been corroborated in previous research where young people claimed they would not use social media [33] or would prefer face-to-face help [51] when going through a difficult time. Further, contrary to evidence suggesting that young people would want to use a self-management tool independently from their parents, our sample considered parental involvement appropriate if needed, a view that is supported by models of young people accessing health care who found parental encouragement helpful [47,52]. Finally, in light of a reported negative relationship between use of social media and well-being levels in youth [53], our young people’s recommendation for use of forums as a communication platform may pose further concerns regarding risks to the young person around privacy or credibility of source.

### Limitations

Although this study helped address a gap in the literature in relation to the elements desired by the youth in a digital technology tool designed for the self-management of well-being, this was a small study with a sample of young people that cannot be regarded as representative of the general youth population. In addition, these findings are limited in breadth given the narrow scope of the participants’ reported digital technology use.

Although participants did not report having received a clinical diagnosis of a mental health problem, the presence and nature of current or previous experience of mental health issues cannot be determined. Moreover, the nature of stressors experienced as well as the presence of significant life events could impact on the use of digital technology tools and the perceived need for help with well-being issues and should therefore be factors for consideration in future explorations.

### Conclusions

The young people’s preferred features and facets in an ideal self-help well-being tool reflected their desire for these elements to be similar to those readily accessible to them through the technology they use on a daily basis for recreational and social purposes. Young people’s desire to use fit-for-purpose and user-informed self-management tools also highlights the need to embed in such tools pointers for directing young people to appropriate mental health information and support.

Such aspects may be important to consider in the refinement of self-help well-being tools with the aim of enhancing their acceptability by the youth population in order to proactively improve psychological well-being levels. However, given the limited scope of this study, these findings warrant further investigation and any conclusions derived from these findings should be tentative.

## Appendix

Multimedia Appendix 1 The semistructured interview guide.

Multimedia Appendix 2 Codebook from Themes 3 and 5 using quotes from participants as examples.
