# Supplementary material for: Desired Features of a Digital Technology Tool for Self-Management of Well-Being in a Nonclinical Sample of Young People: Qualitative Study
Source: JMIR Ment Health. 2018 Dec 18;5(4):e10067. doi: 10.2196/10067 (PMC6315233; doi:10.2196/10067)
Supplement: Multimedia Appendix 1 [file mental_v5i4e10067_app1.pdf]

Appendix 1. The interview guide used in interviews with questions and prompts.

1) What technology do you use?
(Focus on tools used for well-being. E.g. mobile, I-pad, parent's phone, computer)

  i. When do you use it and what do you use it for?
  (Knowledge, organizational, entertaining, social networking, therapeutic)

2) Do you use a tool to help manage your well-being, how does this tool make you feel?
(Explore tool-use: What feelings lead to tool-use/ When used/ How often)

  ii. Do you find the tool helpful? *(Do you use the tool to look after your well-being?)*

3) *WHEN APPROPRIATE:* Have you ever used a mental health service? This can include any times you have spoken to someone about your mental health or well-being?
(e.g. family doctor, nurse, school counsellor, community mental health team, hospital, Childline, therapist)

I'm now going to ask you about a tool that you either already use to look after your mental health and well-being, or if you don't use one can you try and think about a tool that you would use if you were feeling low or unhappy and just imagine that you did use it.
(If they do not understand mental health explain it is the way people feel about themselves, their relationships, and their ability to manage feelings and deal with difficulties are all aspects of mental health)

4) What would be the ideal tool for you if you were having a bad day?
(If you were feeling low, or sad, or stressed)

  i. What would this device be available on?
  ii. What things would you like to see on the tool to help with that?
  (e.g. push notifications, online forum, knowledge, organizational, entertaining/ games, social networking, media, therapeutic)
  iii. What things wouldn't be helpful?

5) If the tool had all these features on it, do you think you would use it?

  i. How often would you use it?
  ii. What would you make you use it more?
  (What parts of the features?)
  iii. What would make you use it less?
  (What parts of the features?)

6) Would anything give you any concerns when using it?
(Privacy, phone space, when downloading, during use)

7) Do you think your parents could play a role in helping you using the self-management tool?
(Is this a tool you would use alongside your parents? Would your parents be involved in a different way, i.e. would they have their own tool?)
