# Supplementary material for: Desired Features of a Digital Technology Tool for Self-Management of Well-Being in a Nonclinical Sample of Young People: Qualitative Study
Source: JMIR Ment Health. 2018 Dec 18;5(4):e10067. doi: 10.2196/10067 (PMC6315233; doi:10.2196/10067)
Supplement: Multimedia Appendix 2 [file mental_v5i4e10067_app2.pdf]

Appendix 2. Table 1. Codebook of theme 3: *The Ideal Tool Should Assess and Direct One to Resources that Match One's Needs*, using quotes from participants as examples.

Theme 3 [T3]: The ideal tool should assess and direct one to resources that match ones needs					
**[T3:1] Provides resources to overcome negative emotions**					
**Participant Number**	8	5	2	4	14
**Quote**	I don't think so, I think it would specify what generally you find takes your stress levels down and it comes up with general subjects that lower your stress levels	So I feel like someone could go on the app and then click on a section depending how they feel, and just put like how long they want to spend on the app or how much they think they need… the app makes a sort of like algorithm formula, and the apps like we don't think you need ten minutes we think you need fifteen. And it's a constant thing of like videos or like a quick games, and words and quotes and like filler things that calm and focus me in that particular section and there can be numerous sections of the app	…like you just tell the app… what you enjoy doing and it kind of picks certain things so you can kind of go, oh get back to what, like if you were stressed out, get back to calm state, just using the app, or tool or whatever and it will kind of uses your information that you've told it to give you something, like a quiz or something just to feel better	Umm well I think the app should just, it should be really motivational, and should be like, oh you need to do maybe 20 minutes of exercise today	Yeah because usually like just in general, if you really don't know what to do, not necessarily just in football but like generally like anything in general, if you don't know what to do afterwards that makes it worse, than if you know what to do. Like say you've got a bad grade or say someone has made fun of you or something like, if you knew how to like, if somethings showing you like okay you need to just like know to ignore that person for a few days, so if you had someone just to show you what to do that would make it better because they can help you see, and help you see that okay this is not really , this is just what's happening now it's not going to be like this forever and it will change
**Code**	Ideal tool - general features - it identifies what reduces your stress levels	Ideal tool - use - person identifies how long they want to spend on the tool, but the app looks at the feelings of the person and makes an algorithm to suggest how long they should spend on the tool, within realms of what the person has said	Ideal tool - general ideas for mood management - the app uses your information to get you back to a calm state, selecting things for you to do	Ideal tool - features - app suggests you get off your phone e.g. go for a walk, do exercise	Instagram - feelings - can make you feel down if someone else looks like they're having a better time than you

**[T3:2] Provides information and direction for further support**					
**Participant Number**	10	8	5	9	5
**Quote**	Umm, advice maybe? You could have some advice on there [If you're feeling stressed]<br>So you'd want some sort of information on there as well?<br>Yeah so if you're going through something bad, something that helps you get through it	Maybe for like, when you're anxious just links to like websites of like, to talk about it	it could just be like, ted talks, I know ted talks do loads of videos about how to cope with that, so it could be more about like self-help and distraction in that sense	Yeah so if it finds out you've got a problem, it then focuses on that and highlights that	Yeah, so I think if someone's very often clicking like low mood and a certain aspect maybe if the app could just like come a bit more focused and talk about, not brainwashing, but develop itself so that the videos it shows could be of someone being like, about them getting help and external forces as well, just like, giving these ideas and showing that there are other ways to do it as well and talking and stuff
**Code**	Ideal tool - relationship struggles - advice and information - could be helpful	Ideal tool - features - signposting - particularly for anxiety or depression	Ideal tool - feature - videos - ted talks good for things like low mood	Ideal tool - tracks mood - negative if it overly focuses on negative moods	Ideal tool - signposting - gives participants more direct information when they show worrying signs

Table 2. Codebook of Theme 5: *Flexibility in Choice and Resources is a Desired Feature in the Ideal Tool*, using quotes from participants as an example.

Theme 5 [T5]: Flexibility in choice and resources is a desired feature in the ideal tool				
**[T5:1] Choice for reflecting different emotional states**				
**Participant Number**	5	13	10	
**Quote**	Inside there are loads of different things for like, different emotions of the person, for like anxiety, stress	Yeah, umm I think the main thing that would interest me with a tool like that is if it gave you choice. It depends on how I'm feeling and what exactly I feel I'm in the mood for to do	Yeah, like an option for how you're feeling and something to help you	
**Code**	Ideal tool - features - lots of things for different emotions	Ideal tool - general function - to be given choice so that depending on your mood you can choose what to do	Ideal tool - general function - an option for a mood and something to help	
**[T5:2] Games and puzzles to suit individual preference**				
**Participant Number**	13	2	8	
**Quote**	Yeah, the only problem with that is that I'd want the games but it would be very difficult for that device or tool to have enough games to fit not only my personality, but a lot of other peoples …ideally you being able to have an infinite supply of different games would be great	Yeah, because I'd be able to feel, especially with like games and bits id be able to feel like, I used or not necessarily used them at the moment, it would help if I was stressed out to take my mind off it and you could like choose what you want or what your favorite kind of thing is, if you like puzzles, or like quizzes or games and bits, you can just choose whatever you want, that would help a lot	Like funny stuff and stuff that makes you think, like mathematical puzzles, like stuff that makes you laugh [would lower your stress levels]<br>Yeah I guess, anything really, like questions that are generally hard to solve which takes your mind off	
**Code**	Ideal tool - games - the tool would need to allow a variation of games to suit peoples personalities or moods, ideally an infinite supply of games	Ideal tool - features - games, puzzles, quizzes which suit lots of people and distract	Ideal tool - features - which make you think and distracts you	
**[T5:3] Videos provide a variety of resources**				

**Participant Number**	5	7	10	8
**Quote**	I think it's hard raising someone's mood without being there yourself so I think like more videos or, this, it could just be one video or maybe 2, it could just be like, ted talks, I know ted talks do loads of videos about how to cope with that, so it could be more about like self-help and distraction in that sense	Like one minute videos and such or like a little extract. because its easy quick and it also means, like it's much easier to understand, it's like quick and to the point and all that	Yeah like when you're on Facebook, and you see a homeless person and that<br><br>Yeah like a minute maybe, something like that	Well it would have to have like funny videos and stuff, and funny pictures …<br>Like memes
**Code**	Ideal tool - feature - videos - ted talks good for things like low mood	Ideal tool - information/ advice - through short videos or reading	Ideal tool - videos - one minute long	Ideal tool - features - funny pictures - like memes to make you laugh
**[T5:4] Music for individual preference**				
**Participant Number**	6	11	13	
**Quote**	Well I'm not quite sure, like I'd like it to be the music that I have, like the music that I play, like quite a lot of guitar stuff would be quite nice. I'm not that bothered by what the music is as long as I enjoy it like I enjoy quite like soft music, I don't like this weird metallic stuff,<br>I don't like that	Um I dunno, probably like different types of music, I dunno like completely different types of music	I certainly would quite like it to be able to play music ad quite a large range to fit what I want, and that would be quite difficult because my music tastes change quite dramatically in a very short amount of time<br>sort of apps like Spotify and then you have a big range of music, they're pretty useful, so you can whatever you really want to listen to you can do that, I think the main thing is that the tool is very personable to what you want to use it	
**Code**	Ideal tool - music - can be any music, would be good if it was own music, but any nice music would work	Ideal tool - feature - music - many completely different types of music to suit mood	Ideal tool - feature - music - many different types of music to suit mood	
